# Aortic dissection presenting as a stroke: a case report

**DOI:** 10.11604/pamj.2023.44.91.38533

**Published:** 2023-02-16

**Authors:** Rafi Daou, Danielle Abou Khater, Rita Khattar, Mariana Helou

**Affiliations:** 1 Department of Emergency Medicine, Lebanese American University Medical Center, Beirut, Lebanon,; 2 Lebanese American University, School of Medicine, Beirut, Lebanon

**Keywords:** Aortic dissection, chest pain, dissection, case report

## Abstract

Aortic dissection is an uncommon yet frequently fatal illness. Patients generally present with tearing chest pain with possible acute hemodynamic instability. Hence, early diagnosis and intervention is critical for survival. This is a case of a 62-year-old male who was transferred to our emergency department for severe chest pain, left side hemiplegia, left hemianopsia, left facial weakness, suggesting a right-sided stroke. A chest computed tomography angiogram showed an extensive circumferential aortic dissection of the intimal layer involving the great vessels. Antiplatelet medications were withheld, nicardipine was started, and the cardiothoracic surgeon was consulted. There was no indication for surgery, and patient was admitted to the intensive care unit. We highlight here the importance of considering an aortic dissection in patients who present with neurological symptoms and an acute history of tearing chest pain.

## Introduction

Aortic dissection (AD) is an uncommon yet frequently fatal illness. Patients generally present with tearing chest pain with possible acute hemodynamic instability. Hence early diagnosis and intervention are critical for survival. Risk factors include advanced age, chronic hypertension, smoking, male gender, atherosclerosis or pre-existing aortic disease like an abdominal aneurysm [1]. Genetic connective tissue diseases such as Marfran, Loeys-Dietz, Ehlers Danlos, or Turner syndrome and bicuspid aortic valves account for the younger population at risk [2]. Pathogenesis involves injury to the tunica intima layer allowing blood to enter the media dissecting the intimal and adventitial layers. This dissection of the layers forms a false lumen which allows blood to flow into. The dissection can extend proximally, distally or in both directions [3].

Aortic dissection can be classified using two different systems, Stanford or DeBakey. With the Stanford classification, type A involves the ascending aorta while type B is limited to the descending aorta only. DeBakey type 1 involves the ascending, arch, and the descending aorta. DeBakey type 2 involves the ascending aorta while type 3 involves the descending aorta only. For diagnosis, the imaging modality of choice is Computed Tomography (CT). CT angiography (CTA) is highly accurate with sensitivity and specificity of 95% and 98% respectively [4]. Its widespread availability, high specificity, and sensitivity make CTA the gold standard for diagnosis and management.

We present below a case presented to the emergency department (ED) for resolving chest pain, left hemiplegia, left hemianopsia, and facial weakness, suspected of having a stroke. Further assessment and workup showed a diagnosis of aortic dissection.

## Patient and observation

**Patient information:** this is a case of a 62-year-old male who was transferred to our ED, which was the closest stroke center to the initial ED he presented at. The report sent with the patient stated that he presented as conscious, and cooperative with left upper and lower extremity hemiplegia. He was phasic and did not have slurred speech. Patient is known to be hypertensive on bisoprolol 5 mg daily and hydrochlorothiazide/irbesartan 300/12.5 mg daily. He was diabetic on metformin 500 mg twice daily and dyslipidemic on simvastatin 10 mg daily. He also had coronary artery disease and had 2 stents placed within the last year and was on aspirin and Clopidorgrel daily. Of note, he also was overweight with a body mass index (BMI) of 31. His vitals recorded at 10:00 were blood pressure (BP) 126/64 mmHg, heart rate (HR) 60/min, saturation O_2_ SpO_2_ 98% on room air, hemoglucotest (HGT) 170 mg/dL. His brain CT report showed a right parietal hypodensity with no hemorrhage which correlated with a right middle cerebral artery (MCA) stroke. He was given antiemetics and an antiepileptic then transferred to our stroke center.

**Clinical findings:** upon arrival to our ED at 14:30 the patient was awake, conscious, and cooperative, left side hemiplegia, left hemianopsia, and left facial weakness, without aphasia or facial drooping. His vital signs were taken: BP 108/51 mmHg, HR 53/min, SpO_2_ 100% on room air, HGT 132 mg/dL, afebrile.

**Timeline of the current episode:** the patient reported experiencing severe tearing chest pain while driving to work the morning of June 8, 2021, at 09:00 am after which he stopped on the side of the road and called his wife due to the chest pain. An ambulance arrived at the scene approximately at 09:45 am and transported the patient to the initial emergency room around 10:00 am with left hemiplegia. Upon arrival at our ED, the patient no longer reported experiencing chest pain.

**Figure 1:**
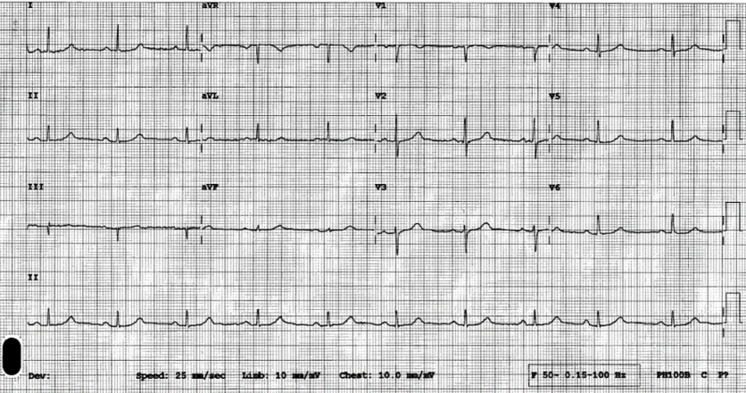
electrocardiogram

**Diagnostic assessment:** electrocardiogram on arrival was normal (Figure 1). A blood test with complete blood count, electrolyte panel, urea, creatinine, cardiac enzymes, and coagulation studies was taken. His cardiac enzymes were elevated; his creatinine was 1.49 mg/dL. A neurological assessment was done and a decision was made to obtain a brain CT and CTA and chest CTA. His chest CTA showed an extensive circumferential aortic dissection of the intimal layer (windsock sign), with the dissection flap extending from the aortic root to the common iliac arteries bilaterally. It appeared to involve the great vessels (right brachiocephalic artery, left common carotid, and left subclavian artery). With also a faint hypodense line seen along the left anterior descending coronary artery (LAD), that could be related to an extension of the dissection flap to involve the LAD. There was associated dilatation of the ascending aorta measuring 5.1 cm in diameter with focal discontinuity of the intimal flap at the level of the lower thoracic aorta and asymmetric enhancement of the kidneys with hypoperfusion of the left one. The celiac trunk, superior mesenteric artery, and inferior mesenteric artery arise from the true lumen and were well opacified.

**Figure 2:**
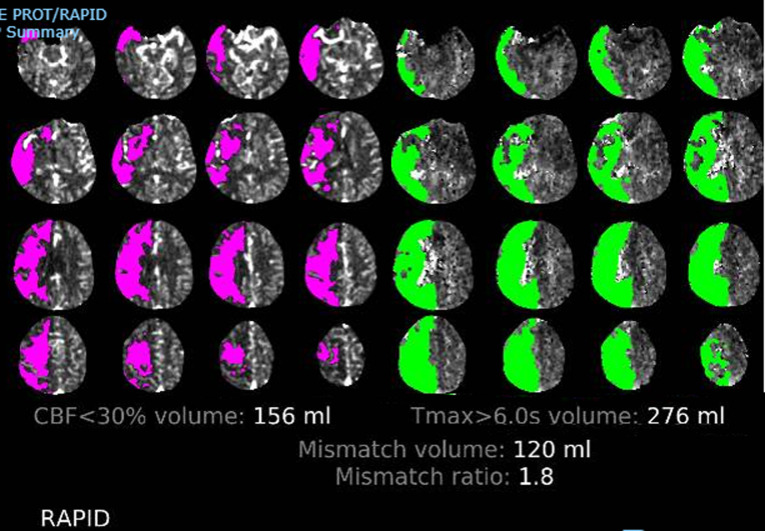
perturbation of the perfusion in the middle cerebral artery territory on brain computed tomographic perfusion sequence

Emergency brain CT scan showed a faintly hypodense area with loss of gray-white matter differentiation almost involving the entire right MCA territory mainly involving the right frontal and parietal lobe, right basal ganglia with effacement of the globus pallidus/insular cortex differentiation as well as the anterior temporal lobe, suggesting acute ischemic insult. Brain CTA showed acute circumferential aortic dissection involving the visualized thoracic aorta (ascending, aortic arch, and descending aorta) with a dissecting flap extending into the right brachiocephalic artery and the left subclavian artery as well as into the common carotid bilaterally with further extension into the proximal portion of the right internal carotid. Brain computed tomographic perfusion sequence (CTP) analysis of the territory of the right MCA showed a perturbation of the perfusion maps with a small viable penumbra and a large core infarct (Figure 2).

**Figure 3:**
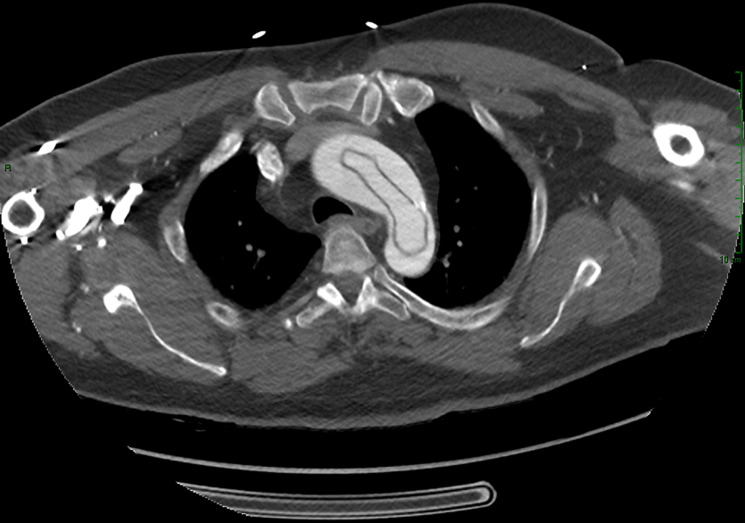
windsock sign: aortic dissection of the intimal layer on chest computed tomography angiogram

**Figure 4:**
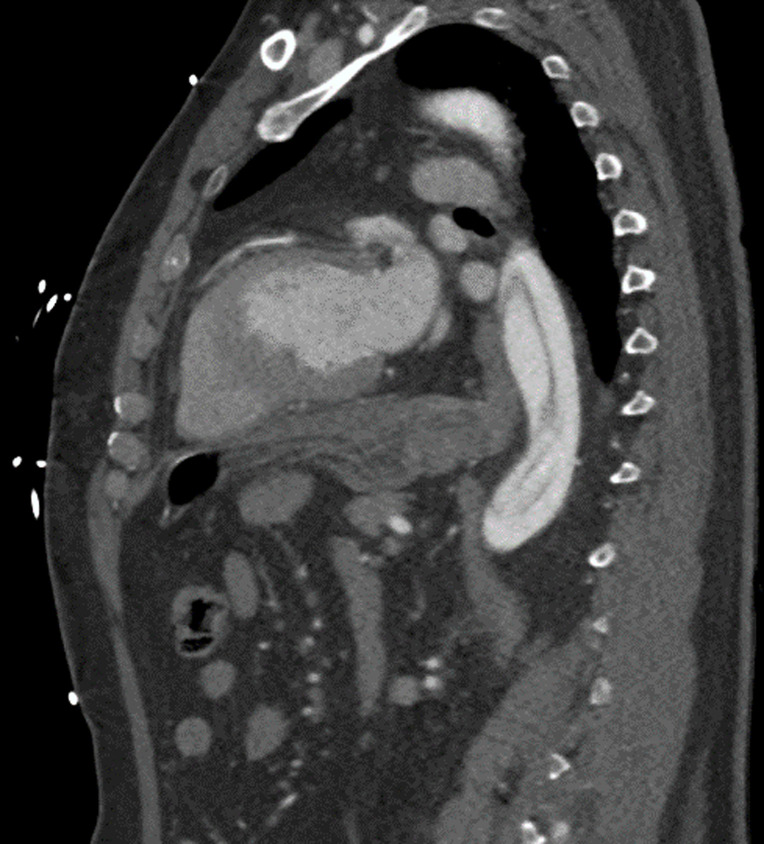
intimal flap on chest computed tomography angiogram

**Diagnosis:** this was consistent with Stanford type A, DeBakey type I aortic dissection (Figure 3, Figure 4).

**Therapeutic interventions:** the antiplatelet medications were withheld and nicardipine was started. The cardiothoracic surgeon decided not to operate due to the high risk of the surgery.

**Follow-up and outcome of interventions:** the patient was admitted to the intensive care unit (ICU). The stay in the ICU was complicated, the creatinine went up to 1.65 mg/dl the next day, and the patient deceased after 3 days.

**Informed consent:** the patient gave his consent upon admission to the emergency.

## Discussion

Our patient had well-known and documented risk factors for developing an acute aortic dissection. He was in the age range being 62-years-old, hypertensive, diabetic, and dyslipidemia. He also had coronary artery disease and was overweight. These risk factors clearly predisposed him to an acute AD. His CT showed no signs of preexisting aortic disease such as an aneurysm, and there was no structural valvular disease seen on his previous transthoracic echocardiogram. The incidence of acute aortic dissection is 2.6 to 3.5 per 100,000 persons per year [5]. A population-based longitudinal study conducted over 27 years found the age range to be 36 to 97 years old with a mean age of 65.7 years [6]. The male/female ratio was 1.55 to 1. They also found a total of 22.7% of the hospitalized patients died within the first 6 hours, 33.3% within 12 hours, 50% within 24 hours, and 68.2% within the first 2 days after admission [6].

Pathogenesis of an acute AD is now better understood. This patient had a clear rapid progression of the dissection. He first reported the chest pain to his wife and by the time he had arrived at the nearest ED he had neurological symptoms with left hemiplegia. He arrived at our ED with left hemiplegia and no chest pain. He reported twitching in his right arm while undergoing imaging at our stroke center. The pathogenesis of the dissection in this patient began with the false lumen appearing in the aortic arch. It dissected anterograde down the descending aorta and retrograde to the LAD and the common carotid artery. The symptoms manifested as neurological, left hemiplegia, and his chest pain was now silent. The question that arises, in this case, was why the patient was no longer experiencing chest pain upon transfer to our hospital considering the extent of the dissection including an extension to the LAD. The patient had described the initial chest pain as tearing in nature, however, as the dissection extended the chest pain became silent, likely the dissection of the LAD did not completely block blood flow. We can postulate that the hypoperfusion to the brain cortex and pain modulators also played a role in silencing the chest pain. Our patient had an acute kidney injury (AKI) with a serum creatinine of 1.49 mg/dL on admission. The mechanisms of an AKI following an acute AD include renal malperfusion or hypoperfusion and underlying kidney disease, which our patient did not have. In a study conducted by Takahashi *et al*. the incidence of AKI was as common as 36%, which also documented that AKI was associated with in-hospital complications such as organ or limb malperfusion, acute lung injury, acute coronary syndrome, and stroke [7]. Our patient´s serum creatinine had risen the next day to 1.65 mg/dL further suggesting the anterograde dissection was the cause of his left renal hypoperfusion seen on CTA.

After establishing the diagnosis, we requested a neurology and cardiothoracic consultation. Management of acute type A aortic dissection consists of replacement of the ascending aorta and “hemiarch” with an open distal anastomosis, together with either aortic valve resuspension and obliteration of the aortic root false lumen or a Bentall´s procedure. The assumption is that this prevents aortic rupture, aortic regurgitation, and coronary ischemia while removing the primary tear and restoring antegrade true lumen perfusion [8]. With our patient the dissection had already reached the LAD, however, we cannot definitively say that the elevated troponin was due to coronary ischemia or his AKI. The cautions and relative contraindications to surgery include cerebrovascular event, severe left ventricular dysfunction, coagulopathy, pregnancy, previous mass index (MI) within the last 6 months, significant arrhythmias, and advanced age. The clear contraindication and decision not to operate were due to the cerebrovascular accident and neurological symptoms.

## Conclusion

We see above the case of a patient with acute AD presenting with neurological symptoms. However, in the absence of chest pain, making the diagnosis can be difficult. Therefore, it is of paramount importance to suspect aortic dissection in any patient presenting chest pain with symptoms of stroke.

## References

[1] Gawinecka J, Schönrath F, von Eckardstein A (2017). Acute aortic dissection: Pathogenesis, risk factors and diagnosis. Swiss Med Wkly.

[2] Poniedzialek-Czajkowska E, Sadowska A, Mierzynski R, Leszczynska-Gorzelak B (2019). Aortic dissection during pregnancy-obstetric perspective. Ginekol Pol.

[3] Tintinalli JE, Ma O, Yealy DM, Meckler GD, Stapczynski J, Cline DM (2020). Tintinallis emergency medicine: A comprehensive study guide, 9e.

[4] Baliga RR, Nienaber CA, Bossone E, Oh JK, Isselbacher EM, Sechtem U (2014). The role of imaging in aortic dissection and related syndromes. JACC Cardiovasc Imaging.

[5] Clouse WD, Hallett JW, Schaff HV, Spittell PC, Rowland CM, Ilstrup DM (2004). Acute aortic dissection: population-based incidence compared with degenerative aortic aneurysm rupture. Mayo Clin Proc.

[6] Mészáros I, Mórocz J, Szlávi J, Schmidt J, Tornóci L, Nagy L (2000). Epidemiology and clinicopathology of aortic dissection. Chest.

[7] Takahashi T, Hasegawa T, Hirata N, Endo A, Yamasaki Y, Ashida K (2014). Impact of acute kidney injury on in-hospital outcomes in patients with DeBakey type III acute aortic dissection. Am J Cardiol.

[8] Matalanis G, Perera NK, Galvin SD (2016). Total aortic repair: the new paradigm in the treatment of acute type A aortic dissection. Ann Cardiothorac Surg.

